# Systematic Review of Patient-Reported Outcome Measures for Patients with Exercise-Induced Leg Pain

**DOI:** 10.3390/medicina58070841

**Published:** 2022-06-23

**Authors:** Alejandro Castillo-Domínguez, Jerónimo C. García-Romero, José Ramón Alvero-Cruz, Tomás Ponce-García, Javier Benítez-Porres, Joaquín Páez-Moguer

**Affiliations:** 1 Department of Nursing and Podiatry, University of Malaga, 29071 Malaga, Spain; joaquinpaez@uma.es; 2 Department of Human Physiology, Histology, Pathological Anatomy and Sports Physical Education, University of Malaga, 29071 Malaga, Spain; jeronimo@uma.es (J.C.G.-R.); alvero@uma.es (J.R.A.-C.); tomas_ponce@uma.es (T.P.-G.); benitez@uma.es (J.B.-P.)

**Keywords:** exercise-induced leg pain, medial tibial stress syndrome, chronic exertional compartment syndrome, stress fracture, running, psychometrics, patient-reported outcome measures

## Abstract

*Background and Objectives*: To determine the most commonly used patient-reported outcome measures (PROMs) in exercise-induced leg pain (EILP) and to identify specific PROMs for EILP in order to evaluate their psychometric properties and methodological quality. *Materials and Methods*: A strategic search was performed in different databases to identify and extract the characteristics of studies based on the use of PROMs in patients with EILP. Specific PROMs were evaluated according to the Terwee et al. and COSMIN criteria. *Results*: Fifty-six studies were included in the review. The Medial Tibial Stress Syndrome Score (MTSSS), Lower Extremity Functional Scale (LEFS) and Exercise-Induced Leg Pain Questionnaire (EILP-Q) were identified as specific PROMs for EILP. The Visual Analog Scale (VAS) was the most widely used instrument in the assessment of EILP. The methodological quality assessment showed six positive values for the LEFS, four for the MTSSS and three for the EILP-Q for the eight psychometric properties analyzed according to the COSMIN criteria. The evaluation of the nine psychometric properties according to Terwee showed five positive values for the LEFS and MTSSS, and three for the EILP-Q. *Conclusions*: The overall methodological quality of the PROMs used was low. The VAS was the most widely used instrument in the assessment of EILP, and the LEFS was the highest quality PROM available for EILP, followed by the MTSSS and EILP-Q, respectively.

## 1. Introduction

Exercise-induced leg pain is a term encompassing painful leg syndromes induced by physical activity and exercise, excluding painful syndromes affecting the thigh, knee, foot and ankle [1]. The main feature of exercise-induced leg pain is the onset of leg pain associated with physical activity, which is pronounced and increases during activity [2] and is relieved by a variable period of rest [3]. Concerning other sporting activities [4], 84.9% of cases of exercise-induced leg pain involve running activities [5]. Despite the wide range of diagnostic possibilities, stress fracture, medial tibial stress syndrome and chronic compartment syndrome are the most common forms of exercise-induced leg pain [3,6,7,8,9].

Their prevalence in the athlete population with chronic leg pain indicates 33% of cases of exercise-induced leg pain as chronic compartment syndrome, 25% as stress fractures and 13% as medial tibial stress syndrome [6]. Based on the clinical practice of different authors, medial tibial stress syndrome is the most common source of lower leg pain, followed by stress fractures and chronic compartment syndrome [3,7]. Symptoms may include warmth, cramping, muscle weakness, paresthesia, numbness, herniation and tightness [10]. Several protocols have also been established to differentiate the various entities of exercise-induced leg pain. These protocols include diagnostic guidelines that associate pain at rest that is sensitive to touch with bone stress injuries (such as stress fractures or medial tibial stress syndrome), the absence of pain at rest that is sensitive to touch with nerve entrapment injuries, and the absence of pain both at rest and to touch with functional popliteal impingement syndrome or chronic compartment syndrome [3].

Patient-reported outcome measurements provide the clinician with qualitative information that complements the clinical examination, optimizing diagnostic and treatment decision-making [11,12]. Different outcome measurement tools have been developed to assess patients with sports-related leg injuries, including the Medial Tibial Stress Syndrome Score (MTSSS) [13], the Lower Extremity Functional Scale (LEFS) [14], the Exercise-Induced Leg Pain Questionnaire (EILP-Q) [15] and the Foot and Ankle Ability Measures (FAAM) [16]. Various studies have recently used patient-reported outcome measures to analyze the severity of symptoms, limitation in function and sports abilities in patients with exercise-induced leg pain [17,18,19,20]. Among the instruments used for the evaluation of patients with exercise-induced leg pain are visual analog scales [21,22,23], generic questionnaires [24,25] and numerical rating scales [26,27]. Both the subjectivity of the construct with respect to symptoms and functional status and the lack of sensitivity to change when applied to the target audience show that these instruments have limitations compared to specific questionnaires [28].

Consequently, in order to determine the acceptability of these questionnaires, certain psychometric properties, such as reliability and validity, must be met [29]. Reliability has traditionally been defined as the degree to which an instrument is free of random error [30] and is assessed through internal consistency, temporal stability and inter-observer agreement [31,32]. Face validity indicates whether the instrument measures what it intends to measure in the setting it is designed to be applied and it is determined subjectively by the experts [33,34]. Content validity assesses whether the instrument items represent the concepts they are intended to measure and reflects the extent to which the instrument samples all dimensions of the appropriate construct indicators [35]. Construct validity compares equal factors of the instrument used with another instrument having the same factor [36]. As part of construct validity, factor validity is used to establish the scale’s factor structure. Criterion validity analyzes how the instrument being assessed correlates with other similar instruments that have already been validated, assessing the relationship of the new measure with a gold standard [37]. The minimum detectable change shows what changes are outside the error of health status measurement (either based on internal reliability or test-retest in stable individuals). It indicates the difference in score needed to declare a detectable change between repeated measures. However, it is a statistical threshold because the minimally important difference is more appropriate than the minimum detectable change for decision-making in a clinical context [38].

A number of reviews have addressed the diagnosis, evaluation or management of both exercise-induced leg pain and the different conditions involved [1,39,40,41,42]. However, to our knowledge, no reviews have been performed on the patient-reported outcome measures used to evaluate patients with exercise-induced leg pain. For this reason, the objectives of this study were to determine the frequency and characteristics of the use of patient-reported outcome measures in exercise-induced leg pain and to identify the specific instruments in order to evaluate their psychometric properties and methodological quality.

## 2. Materials and Methods

A systematic review was performed based on a pre-specified protocol (CRD42021229309) in accordance with the recommendations of the Preferred Reporting Items for Systematic Reviews and Meta-Analyses (PRISMA) statement [43] and registered in PROSPERO.

### 2.1. Data Sources and Searches

Electronic searches were conducted from the database start date to January 2021, and an updated search (including in-process and non-indexed citations) was completed in March 2022. Studies were selected for analysis, according to the PRISMA guidelines [44], based on searching the following databases: MEDLINE, CINAHL, SPORTDiscus and Cochrane Library.

The search strategy was performed similar to that described by Terwee et al. [45] to obtain the psychometric properties of the instruments, including construct search (patient-reported outcomes specific to exercise-induced leg pain), population search (subjects with exercise-induced leg pain) and instrument search (questionnaires, tests, or scales). The following terms were used, linked through the operators “OR” and “AND”: exercise induced leg pain, chronic exertional compartment syndrome, medial tibial stress syndrome, popliteal artery entrapment, nerve entrapment, stress fracture, patient-reported outcomes, leg, lower leg, chronic pain, soreness and overuse injuries (Appendix A).

### 2.2. Study Selection

The inclusion criteria for the studies were: (a) Studies with participants presenting exercise-induced leg pain (medial tibial stress syndrome, stress fracture, chronic compartment syndrome, popliteal entrapment syndrome or nerve entrapments) over 18 years of age; (b) Studies on psychometric validations of patient-reported outcome measures; (c) Original research without language restrictions using these instruments to assess patients before or after applying a leg intervention in a clinical setting.

The type of results (outcome) included were: (a) Psychometric or clinimetric properties based on the criteria of Terwee et al. [29] (content validity, internal consistency, criterion validity, construct validity, reproducibility, reliability, responsiveness, ceiling/floor effect and interpretability); (b) Psychometric or clinimetric properties according to the COnsensus-based Standards for the selection of health Measurement Instruments (COSMIN) criteria [46] (structural validity, internal consistency, reliability, measurement error, hypothesis testing for construct validity, cross-cultural validity, criterion validity and responsiveness).

The exclusion criteria were: (a) Studies that used questionnaires without evidence of reliability or validation; (b) Studies that were not focused on exercise-induced leg pain, systematic reviews, comments to the editor and case reports were excluded from the study. No restrictions on publication date were imposed.

Two blinded reviewers (A.C.D.-J.P.M.) evaluated the search results. The list of studies was reviewed independently to ensure that the inclusion criteria were met. Disagreements were resolved by discussion between the two reviewers. If consensus was not reached, the opinion of a third or fourth reviewer (J.C.G.R.-J.R.A.C.) was sought. The study selection process was carried out using the Web App Rayyan [47], developed to facilitate screening, scanning, filtering and elimination of duplicate studies.

### 2.3. Data Extraction

Data extraction was related to the title of the outcome measure used, number of scale items, number of dimensions, score ranges, interpretation of the results, population for which it is validated, number of studies and total number of patients assessed by each outcome measure. The following data were also extracted for studies on the validation of specific patient-reported outcome measures: psychometric properties according to the Terwee et al. criteria [29], methodological quality according to COSMIN [46] and cross-cultural adaptations of each questionnaire to different languages.

### 2.4. Quality Assessment

To assess the methodological quality of the research studies on the measurement properties of patient-reported outcome measures, the updated COSMIN checklist was used [46]. This method can be used both to assess the methodological quality of studies of patient-reported outcome measures [11] and to compare the measurement properties of several instruments in a systematic review [48]. In addition, the studies were evaluated according to the psychometric properties published by Terwee et al. [29].

### 2.5. Data Synthesis and Analysis

For COSMIN checklist, measurement properties were considered in relation to three domains: reliability, validity and responsiveness. The “worst score counts” approach was applied to obtain the final patient-reported outcome measure rating. Each property contains several items assessed as poor, fair, good or excellent by specific criteria that are described in the COSMIN checklist on a 4-level Likert [48].

The following psychometric properties were scored based on the criteria described by Terwee et al. [29]: content validity, internal consistency, criterion validity, construct validity, reproducibility (agreement and reliability), responsiveness, floor/ceiling effects and interpretability. Each aspect was rated as positive “+” (adequate description or value or measure or argument related to the psychometric property), negative “-” (inadequate or values below accepted norms in each psychometric property), indeterminate “?” (questionable method, measure, or design) or absent “0” (no information available on a psychometric property), with the exception of responsiveness, which was scored only as present/absent.

## 3. Results

**Figure 1:**
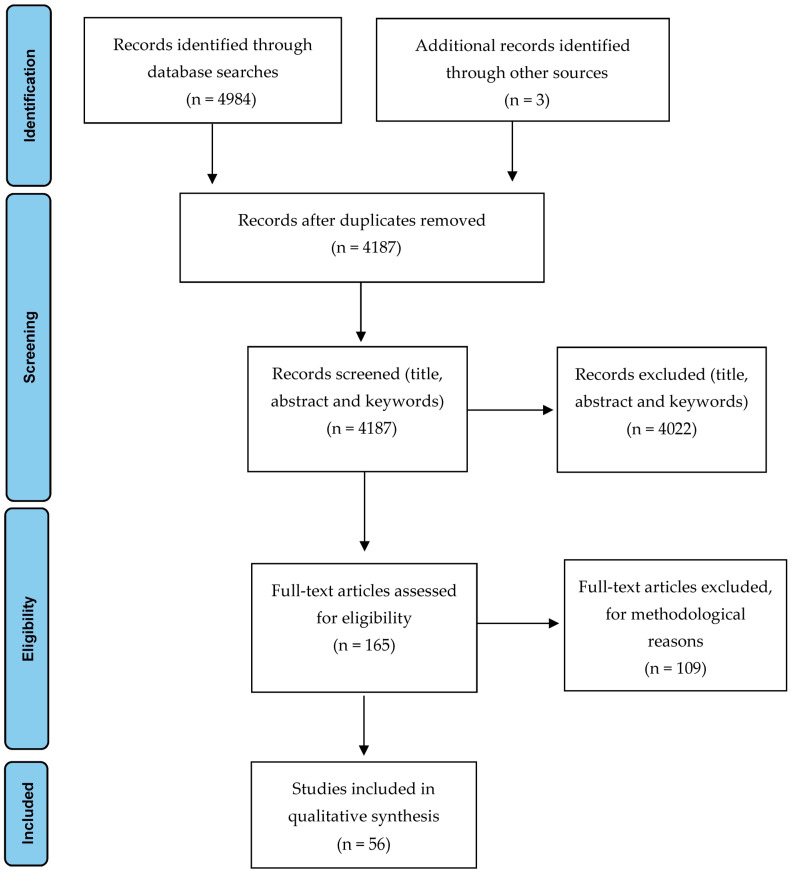
PRISMA Flow Diagram [44]. For more information, visit www.prisma-statement.org accessed on 26 May 2022.

We identified 4984 potential studies, of which 797 were duplicate studies in different databases. Through the references of the included studies, a total of three studies were located and manually added. The remaining 4187 were screened according to the inclusion/exclusion criteria, using titles, abstracts and keywords. This process led to the exclusion of 4022 studies, in most cases because they were not psychometric validation studies of patient-reported outcome measures or because they did not focus on exercise-induced leg pain. The application of the quality assessment filter resulted in the exclusion of 107 additional studies, excluding studies that did not use valid patient-reported outcome measures, systematic reviews, comments to the editor and case reports. After a detailed reading of the remaining 58 articles, a further two were excluded, with 56 being included for the final review. Figure 1 shows the PRISMA flow diagram for the studies included in this review.

A total of 18 outcome tools that were used to assess assessing exercise-induced leg pain were identified from the initial database search. The psychometric properties of the validation studies of three exercise-induced leg pain-specific patient-reported outcome measures were analyzed and reviewed. A summary of the ten most commonly used scoring systems (generics and specifics) can be found in Table 1, including title, the number of items, dimensions, score ranges, how to interpret the results and the population for which they are validated.

### 3.1. Specific Patient-Reported Outcome Measures for Exercise-Induced Leg Pain

After a detailed examination of 56 full-text articles, three validation studies of patient-reported outcome measures specific to exercise-induced leg pain (MTSSS, EILP-Q and LEFS) were included in the review. These instruments were homogeneous in terms of the number of dimensions, and somewhat less so in terms of the number of items. The latter ranged from four in the MTSSS questionnaire to 20 in the LEFS scale. The areas addressed in the studies included symptom severity (limitation of physical activity, pain at rest, in daily activities and during sports practice), physical function (quality of life, limitation in household chores and general leg health) and athletic ability (limitation of movements associated with a sports movement and restriction of athletic and recreational activity or function). Regarding the number of items included, the patient-reported outcome measures ranged from long versions with 20 items in the LEFS [14], to only four items in the MTSSS [13].

### 3.2. Psychometric Properties

The psychometric properties considered according to the Terwee et al. criteria [29] for each instrument are summarized in Table 2.

#### 3.2.1. Content Validity

All the patient-reported outcome measures provided a clear description of the purpose of the measurement and the target population, defining the criteria for item selection and exclusion. In the EILP-Q, MTSSS and LEFS, the target population was included during item selection, as were researchers and experts. All these patient-reported outcome measures provided details on the interpretability of the items, although this is not an essential characteristic for content validity.

#### 3.2.2. Internal Consistency

Internal consistency was evaluated using Cronbach’s alpha for the entire instrument. The EILP-Q obtained a positive rating, with a value (α = 0.924) ranging from 0.7 to 0.95. The LEFS had a negative rating, with a value (α = 0.96) greater than 0.95. The MTSSS scale had a negative rating, with a value (α = 0.58) less than 0.7.

#### 3.2.3. Criterion Validity

None of the instruments obtained a positive rating for criterion validity, which required a strong correlation >0.7 with the gold standard. The MTSSS scale had a negative rating, with a weak correlation. The LEFS questionnaire provided no information regarding the gold standard, and the EILP-Q had deficiencies in its methodology compared to the gold standard.

#### 3.2.4. Construct Validity

MTSSS and LEFS were positively rated. Both specified the hypotheses in advance and at least 75% of the results corresponded with these hypotheses (groups ≥ 50 patients). The EILP-Q was rated negatively because no specific hypotheses were formulated in advance, although this criterion was not absent.

#### 3.2.5. Reproducibility Agreement

MTSSS and LEFS had a positive rating for reproducibility agreement. In both, the minimal important change was defined and, despite obtaining the same values as the smallest detectable change, agreement was supported on the basis of the authors’ experience with the interpretation of the questionnaire scores. For the EILP-Q, this information was not available.

#### 3.2.6. Reproducibility Reliability

The MTSSS, LEFS and EILP-Q obtained a positive value for reproducibility reliability, with an intraclass correlation coefficient greater than 0.7 and samples with more than 50 subjects.

#### 3.2.7. Responsiveness

All the patient-reported outcome measures provided information on the smallest detectable change, but in all cases, either the methodology applied was questionable or no evidence of a clinically important change was presented.

#### 3.2.8. Ceiling and Floor Effects

Ceiling and floor effects were only described for the MTSSS and the LEFS, with no ceiling or floor effects in samples of more than 50 subjects. The EILP-Q did not provide information in this regard.

#### 3.2.9. Interpretability

The LEFS and MTSSS scales defined the minimal detectable change. However, they did not include information to help interpret the scores in different subgroups (such as the general population) and were therefore classified as “indeterminate”. The EILP-Q did not define the minimal detectable change and was classified as “indeterminate”.

#### 3.2.10. Cross-Cultural Adaptation

The patient-reported outcome measures varied widely in cross-cultural adaptation. The MTSS showed only one adaptation to another language (English). The EILP-Q showed four adaptations to other languages (Spanish, English, French and Greek), and the LEFS instrument has been adapted to eight different languages (Italian, Dutch, Spanish, Greek, Brazilian, Arabic, Chinese and Iranian).

### 3.3. Methodological Quality

The LEFS scale obtained the best results in terms of methodological quality, according to the COSMIN criteria. This instrument obtained positive scores for internal consistency, reliability, measurement error, hypothesis testing for construct validity, cross-cultural validity and responsiveness. The LEFS obtained indeterminate values for structural validity and criterion validity. The next best performing instruments in this regard were the MTSSS and the EILP-Q, which obtained positive values for four and three criteria, respectively (Table 3). 

#### 3.3.1. Structural Validity

None of the patient-reported outcome measures obtained a positive value for structural validity. Insufficient information was provided and a negative rating was obtained.

#### 3.3.2. Internal Consistency

All the instruments except the MTSSS had a positive internal consistency score, for which Cronbach’s alpha was less than 0.70. The EILP-Q and the LEFS had Cronbach’s alpha values greater than 0.70. In this case, the criterion of Cronbach’s alpha < 0.95 was eliminated, since it consisted of the evaluation of an existing patient-reported outcome measure.

#### 3.3.3. Reliability

For reliability, all the patient-reported outcome measures obtained an ICC > 0.70, thus receiving a positive rating, with an interval of 7 to 10 days.

#### 3.3.4. Measurement Error

For the LEFS and MTSSS scales, the minimal detectable change was defined, and the smallest detectable change values were not greater than the minimal detectable change, resulting in a positive rating. The EILP-Q was classified as “indeterminate” for measurement error, since it did not define the minimal detectable change.

#### 3.3.5. Hypothesis Testing for Construct Validity

For the MTSSS and LEFS scales, a study hypothesis was defined and corroborated by the results obtained. Both scales therefore received a positive score. For the EILP-Q, no prior hypotheses were posed, and thus it received an “indeterminate” rating.

#### 3.3.6. Cross-Cultural Validity/Measurement Invariance

Only EILP-Q and LEFS scored positively for this property. The remaining MTSSS was scored as indeterminate because no studies were found with which to compare differences between group characteristics or item functioning.

#### 3.3.7. Criterion Validity

None of the patient-reported outcome measures received a positive criterion validity rating, receiving an “indeterminate” rating due to the lack of use of a gold standard tool during the development of the instrument. For the EILP-Q, criterion validity was assessed using the established, but not validated, postsurgical classification system for chronic compartment syndrome [49].

#### 3.3.8. Responsiveness

LEFS and MTSSS were positively rated for responsiveness, as the results obtained were consistent with the study hypothesis. Only the EILP-Q scored negatively, with results not consistent with the hypothesis.

### 3.4. Methodological Quality Scores for Measurement Properties in Each Study

The methodological quality ratings are summarized in Table 4. EILP-Q, MTSSS and LEFS obtained more positive than negative values and were therefore eligible for evaluation. However, analysis of the methodological quality scores for the measurement properties in each study showed that none were of excellent quality. The overall level of quality of the patient-reported outcome measures considered was low. The MTSSS obtained the best score in this section, with excellent ratings for structural validity and content validity, good ratings for internal consistency, hypothesis testing and responsiveness, and poor ratings for measurement error, reliability and criterion validity. None of the patient-reported outcome measures were assessed for cross-cultural validity, since the inclusion criteria limited the studies to the exercise-induced leg pain context.

### 3.5. Frequency of Patient-Reported Outcome Measure Use in Exercise-Induced Leg Pain

The following 10 outcome measurement tools were the most frequently encountered in this review, listed from highest to lowest frequency: Visual Analog Scale (VAS), 27 articles; Exercise-Induced Leg Pain Questionnaire (EILP-Q), 7 articles; Medial Tibial Stress Syndrome Score (MTSSS), 7 articles; Numerical Pain Rating Scale-11 (NPRS-11), 5 articles; Lower Extremity Functional Scale (LEFS), 5 articles; Foot and Ankle Ability Measures (FAAM), 4 articles; Single Assessment Numeric Evaluation (SANE), 4 articles; Short Form-36 (SF-36), 4 articles; Short Form-12 (SF-12), 3 articles; and Verbal Rating Scale (VRS), 3 articles.

**Figure 2:**
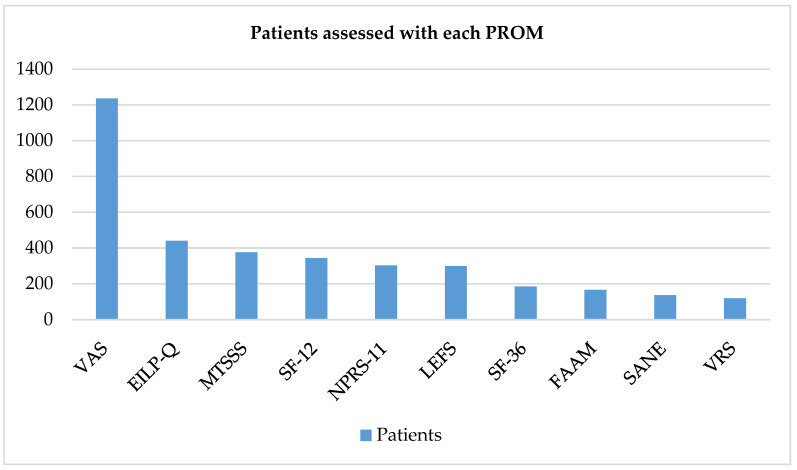
Number of patients assessed with each PROM.PROM = Patient-Reported Outcome Measures; VAS = Visual Analog Scale; MTSSS = Medial Tibial Stress Syndrome Score; EILP-Q = Exercise-Induced Leg Pain Questionnaire; NPRS-11 = Numerical Pain Rating Scale-11; LEFS = Lower Extremity Functional Scale; FAAM = Foot and Ankle Ability Measures; SANE = Single Assessment Numeric Evaluation; SF-12 = Short Form-12; SF-36 = Short Form-36 (SF-36) and VRS = Verbal Rating Scale.

The trend in the number of patients evaluated by each scoring system followed a similar pattern. The VAS was used to evaluate 1235 patients, MTSSS for 376, EILP-Q for 440, NPRS-11 for 303, LEFS for 299, FAAM for 166, SANE for 136, SF-12 for 344, SF-36 for 185 and VRS for 119 patients (Figure 2). The VAS, EILP-Q and MTSSS were, in this order, the three most frequently used systems, both in terms of the number of articles in which they appear and the number of patients included in the assessment.

## 4. Discussion

This review showed that the largest proportion of instruments used in evaluating the exercise-induced leg pain corresponded to generic patient-reported outcome measures, with the VAS predominating as the most widely used instrument both in a number of studies and number of patients. Among the studies analyzed, three specific patient-reported outcome measures validated for conditions or regions associated with exercise-induced leg pain were found (MTSSS, EILP-Q and LEFS).

A systematic review by Shazadeh-Safavi et al. on patient-reported outcome measures in the foot and ankle [50] also found the VAS to be the most frequently used validated instrument both in studies and in patients with foot and ankle disorders. The main disadvantage of generic patient-reported outcome measures for assessing a specific region or condition is their low sensitivity and specificity of constructs compared to specific instruments [28,29]. The VAS is a valid, reliable and reproducible generic patient-reported outcome measure for pain and function that assesses the patient’s perception of pain [51]. Although it has low specificity, it is a highly accepted and validated scale for musculoskeletal conditions [51,52,53]. A reduction in VAS of 30 mm is the minimum clinically important difference in pain severity associated with adequate pain improvement [54], and this is the smallest score change that corresponds to an actual change in the functional condition or status of a patient [55].

The specific instrument that provided the best psychometric properties based on COSMIN was the LEFS. This self-administered, anatomical region-specific questionnaire was initially developed and validated to assess levels of lower extremity disability [14]. This scale has more items than the EILP-Q and the MTSSS but coincides in the number of dimensions. The LEFS comprises 20 questions pertaining to the ability to perform functional activities of daily living. Its validity, reliability and responsiveness have been demonstrated in a wide variety of populations with hip [56], ankle [57], knee [58] and exercise-induced leg pain [19,59]. Each item is scored on a scale from 0 (extreme difficulty or disability) to 4 (no difficulty), for a maximum total score of 80. The score obtained on the LEFS can partially predict the time to complete recovery in patients with MTSS, with poorer scores associated with an increase in the number of days to total recovery from this condition included within exercise-induced leg pain [60]. The LEFS has been translated, cross-culturally adapted and successfully validated in several languages: Italian [61], French [62], German [63], Spanish [64], Greek [65], Portuguese [66], Arabic [67] and Chinese [68], among others [69,70,71,72]. However, none of these studies included participants with exercise-induced leg pain. Considering these characteristics and based on the psychometric properties studied so far, the authors recommend using the LEFS to assess exercise-induced leg pain.

The MTSSS questionnaire provided the second-best psychometric properties based on COSMIN. In addition, MTSSS and LEFS performed best according to the Terwee et al. criteria, although the number of positives was lower compared to the COSMIN criteria. The MTSSS scale has not been successfully adapted and validated in languages other than the original German version. This could be a limitation for its use in other populations. The EILP-Q, originally developed in German, has been adapted and validated in French [37], Greek [73] and Spanish [74]. However, its psychometric properties, according to COSMIN and Terwee et al’s, criteria were the lowest, suggesting the need for further evaluations of its responsiveness, interpretability, criterion validity and ceiling and floor effects.

Limitations of this study are inherent to the design of the review (i.e., language restrictions) and to the methodological quality and weaknesses of the studies included (sample size, heterogeneity, lack of gold standard for evaluating criterion validity, etc.) [75]. The limited number of specific instruments designed for patients with exercise-induced leg pain conditions prevented a more significant number of tools from being compared.

The main strength of this study is the rigorous method developed for the systematic review, which included a blinded peer review of quality appraisal using a standard procedure (COSMIN) and a detailed process for finding studies and specific instruments. This review provides useful information for researchers, trainers and clinicians regarding the patient-reported outcome measures developed and used for patients with exercise-induced leg pain. With high incidence rates [6] and complex treatment pathways [3], tools that can capture the impact of exercise-induced leg pain are essential. Patient-reported outcome measures can help achieve this goal by examining the efficacy of interventions, assessing the evolution of conditions and contributing to shared decision-making between patients and therapists. We recommend that instruments that present poor evidence of their psychometric properties should be used with caution.

More studies are needed in this field to reduce the limitations observed in the specific instruments examined in this study, perhaps by focusing on the use of those designed specifically for the assessment of conditions encompassed within exercise-induced leg pain and considering the COSMIN criteria of those that obtained the highest scores in our review. Furthermore, the psychometric properties and methodological quality of the successive adaptations should be evaluated to better inform clinicians about these self-report assessment instruments for exercise-induced leg pain.

## 5. Conclusions

The VAS was the most frequently used instrument in the assessment of exercise-induced leg pain, and the LEFS, EILP-Q and MTSSS are specific patient-reported outcome measures shown to be promising alternatives to the generic instruments in use. The analysis of the methodological quality concluded that further studies are needed to more fully evaluate the psychometric properties of these instruments, considering the overall low methodological quality of the instruments considered. We found the LEFS questionnaire to be the most suitable specific patient-reported outcome measure currently available for the assessment of exercise-induced leg pain.

## Figures and Tables

**Table 1:** Characteristics of the 10 most commonly used instruments in the assessment of patients with exercise-induced leg pain.

Acronym	Full Title	Number of Items	Dimensions (Items)	Score Range	Interpretation of Score	Type of Population It Validates
VAS	Visual Analog Scale	20	Pain (4), function (11) and others (5)	0–100 mm	Longer distance indicates poorer outcome	Generic. Not specific to a disease or region
MTSSS	Medial Tibial Stress Syndrome Score	4	Severity of medial tibial stress syndrome (4)	0–10 points	Lower score indicates better outcome	Medial tibial stress syndrome
EILP-Q	Exercise-Induced Leg Pain Questionnaire	10	Physical function and athletic ability (10)	0–40 points	Higher score indicates better outcome	Patients with exercise-induced leg pain
NPRS	Numerical Rating Scale-11	11	Pain (11)	0–10 points	Higher score indicates greater pain intensity	Patients with rheumatic pain and other chronic conditions (pain > 6 months)
LEFS	Lower Extremity Functional Scale	20	Physical function (20)	0–80 points	Higher score indicates better outcome	Adults with lower extremity dysfunction
FAAM	Foot and Ankle Ability Measure	29	Activities of daily living (21) and physical function (8)	0%–100%	Higher percentage indicates better outcome	Chronic ankle instability
SANE	Single Assessment Numeric Evaluation	1	Overall function rating	0–100 points	Higher score indicates better outcome	Patients with foot and ankle pathology
SF-12	Short Form-12	12	Physical component (12) and mental component (12)	0–100 points for each component	Higher score indicates better outcome	Generic. Not specific to a disease or region
SF-36	Short Form-36	36	Physical function (10), physical role (4), bodily pain (2), general health (5), vitality (4), social function (2), emotional role (3), mental health (5) and health changes (1)	0–100 each component (physical and mental)	Higher score indicates better outcome	Generic. Not specific to a disease or region
VRS-7	Verbal Rating Scale	7	Pain (7)	1–7 points	Higher score indicates poorer outcome	Generic. Not specific to a disease or region

**Table 2:** Summary of the assessment of the measurement properties of the specific questionnaires described by Terwee et al. [29].

	Content Validity	Internal Consistency	Criterion Validity	Construct Validity	Reproducibility Agreement	Reproducibility Reliability	Responsiveness	Floor and Ceiling Effects	Interpretability
MTSSS	+	−	−	+	+	+	−	+	?
EILP-Q	+	+	?	−	0	+	0	0	0
LEFS	+	−	0	+	+	+	?	+	?

Rating: + Positive rating; ? Indeterminate rating; − Negative rating; 0 No information available. MTSSS = Medial Tibial Stress Syndrome Score; EILP-Q = Exercise-Induced Leg Pain Questionnaire; LEFS = Lower Extremity Functional Scale.

**Table 3:** Detailed COSMIN ratings [46].

	Structural Validity	Internal Consistency	Reliability	Measurement Error	Hypothesis Testing for Construct Validity	Cross-Cultural Validity	Criterion Validity	Responsiveness
MTSSS	−	−	+	+	+	?	?	+
EILP-Q	−	+	+	?	?	+	?	?
LEFS	−	+	+	+	+	+	?	+

Rating: + Positive rating; ? Indeterminate rating; − Negative rating. MTSSS = Medial Tibial Stress Syndrome Score; EILP-Q = Exercise-Induced Leg Pain Questionnaire; LEFS = Lower Extremity Functional Scale.

**Table 4:** COSMIN methodological quality scores for measurement properties of PROMs * [46].

	BOX A Internal Consistency	BOX B Reliability	BOX C Measurement Error	BOX D Content Validity	BOX E Structural Validity	BOX F Hypothesis Testing	BOX G Cross-Cultural Validity	BOX H Criterion Validity	BOX I Responsiveness
MTSSS	GOOD	POOR	POOR	EXCELLENT	EXCELLENT	GOOD		POOR	GOOD
EILP-Q	POOR	POOR	FAIR	EXCELLENT	POOR	POOR		POOR	POOR
LEFS	FAIR	POOR	FAIR	EXCELLENT	POOR	FAIR		POOR	FAIR

* Each property was assessed on a 4-level Likert scale as poor, fair, good or excellent. PROMs = Patient-Reported Outcome Measures; MTSSS = Medial Tibial Stress Syndrome Score; EILP-Q = Exercise-Induced Leg Pain Questionnaire; LEFS = Lower Extremity Functional Scale.

## Data Availability

Not applicable.

## Appendix A. Search Strategy

“Exercise induced leg pain”[tiab] OR “exercise related leg pain”[tiab] OR “chronic exertional compartment syndrome”[tiab] OR “anterior compartment”[tiab] OR “posterior compartment”[tiab] OR “peroneal compartment”[tiab] OR “exertional leg pain”[tiab] OR “anterior compartment syndrome/complications”[Mesh] OR “anterior compartment syndrome/diagnosis”[Mesh] OR “anterior compartment syndrome/therapy”[Mesh] OR “compartment syndromes/diagnosis”[Mesh] OR “compartment syndromes/surgery”[Mesh] OR “compartment syndromes/therapy”[Mesh] OR “fasciotomy”[Mesh] OR “tibia/injuries”[Mesh] OR “medial tibial stress syndrome”[Mesh] OR tibial stress syndrome*[tiab] OR shin splint*[tiab] OR shin soreness*[tiab] OR tibial stress injur*[tiab] OR shinsplint*[tiab] OR shin splint syndrome*[tiab] OR medial tibial syndrome*[tiab] OR shinsplint*[tiab] OR “tibial pain”[tiab] OR “shin pain”[tiab] OR periostalgia[tiab] OR periostitis[tiab] OR traction[tiab] OR popliteal artery entrapment[tiab] OR FPAES[tiab] OR “Nerve Compression Syndromes”[Mesh] OR “Peroneal Nerve/injuries”[Mesh] OR “Sural Nerve/injuries”[Mesh] OR ”Tibial Nerve/injuries”[Mesh] OR Nerve entrapment*[tiab] OR “Fractures, Stress/complications”[Mesh] OR “Fractures, Stress/diagnosis”[Mesh] OR “Fractures, Stress/therapy”[Mesh] OR “tibial stress fracture”[tiab] OR “medial tibial stress fracture”[tiab] AND “patient reported outcomes”[tiab] OR instrument[tiab] OR questionnaire[tiab] OR index[tiab] OR inventory[tiab] OR scale[tiab] OR score[tiab] OR psychometric*[tiab] OR reliability[tiab] OR measure*[tiab] OR validity[tiab] OR inventory*[tiab] OR “psychometric properties”[tiab] OR “factor analysis”[tiab] OR alpha[tiab] AND “lower leg pain”[tiab] OR “leg pain”[tiab] OR “lower leg pain”[tiab] OR “lower extremity pain”[tiab] OR “chronic leg pain”[tiab] OR “lower limb pain”[tiab] OR “jogging/injuries”[Mesh] OR “athletic injuries”[Mesh] OR “overuse injuries”[tiab] OR leg Injuries/complications[Mesh] OR leg Injuries/diagnosis[Mesh] OR Leg Injuries/therapy[Mesh] OR leg injury[tiab] OR leg injuries[tiab] OR shin injuries[tiab] OR lower leg injury[tiab] OR lower leg injuries[tiab] OR lower extremity injury[tiab] OR lower extremity injuries[tiab] OR lower limb injury[tiab] OR lower limb injuries[tiab] OR leg soreness[tiab] OR lower extremity stress[tiab] OR lower limb stress[tiab] OR “leg”[MeSH Terms] OR “leg”[All Fields] OR “lower extremity”[MeSH Terms] OR “lower extremity”[All Fields] OR “lower leg”[MeSH Terms] OR “lower leg”[All Fields] OR “lower limb”[MeSH Terms] OR “lower limb”[All Fields] OR “tibia”[MeSH Terms] OR “tibia”[All Fields] OR “fibula”[MeSH Terms] OR “fibula”[All Fields] OR “patella”[MeSH Terms] OR “patella”[All Fields].
